# Antimicrobial and Cytotoxic Activities of* Turbinariaconoides* (J.Agardh) Kuetz

**Published:** 2010

**Authors:** Sadish Kumar Shanmugam, Yatendra Kumar, Khan Mohammad SardarYar, Vivek Gupta, Erik De Clercq

**Affiliations:** a *Department of Pharmaceutical Chemistry, I.T.S.Paramedical College (Pharmacy), Ghaziabad, Uttar Pradesh, India.*; b *Department of Pharmaceutical Chemistry, JamiaHamdard, New Delhi, India.*; c *Department of Pharmaceutics, I.T.S.Paramedical College (Pharmacy), Ghaziabad, Uttar Pradesh, India.*; d * Rega Institute for Medical Research, KatholiekeUniversiteit Leuven, Minderbroedersstraat 10, B-3000, Leuven, Belgium.*

**Keywords:** Brown alga, *Turbinariaconoides*, Antibacterial, Antifungal, Antiviral, Cytotoxicity

## Abstract

Brown alga, *Turbinariaconoides*was successively extracted with *n*-hexane, cyclohexane, methanol and ethanol:water (1:1). The extracts were evaluated for antibacterial and antifungal activities by disc diffusion method. Minimal inhibitory concentration was determined for active extracts by broth dilution method. The antiviral activity and cytotoxicity of the extracts were tested in human embryonic lung (HEL) cells (herpes simplex virus-1, herpes simplex virus-2, vaccinia virus, vesicular stomatitis virus and herpes simplex virus-1 TK- KOS ACVr), human epithelial (HeLa) cells (vesicular stomatitis virus and coxsackie virus B4) and Vero cells (parainfluenza-3 virus, reovirus-1, sindbis virus coxsackie virus B4 and puntatoro virus). The results revealed that extracts exhibited cytotoxicity ranged from 20 to >100 μg/mL. Moderate activity was demonstrated by *n*-hexane and cyclohexane extracts against viruses, whereas methanol and ethanol:water (1:1) extracts were not active. Ethanol:water (1:1) presented neither antibacterial nor antifungal activity against tested organisms. Cyclohexane extract possessed a broad array of antibacterial activity and exhibited remarkable antifungal property. It is noteworthy that minimal inhibitory concentration of cyclohexane extract against *Aspergillusniger*is comparable with that of clotrimazole. This potentiality demonstrates that it could be used to treat bacterial and fungal infections.

## Introduction

Marine Brown alga, *Turbinariaconoides*belongs to the Sargassaceae family. Turbinaria and other members of the family Sargassaceae are inedible, due to the concentration of polyphenolic substances based upon the polymerization of phloroglucinol (1, 2). Oxygenated steroids of algae have been shown to exhibit cytotoxic properties (3-8). The ethyl acetate extract of *Turbinariaconoides*and its oxygenated fucosterols has been reported for their cytotoxicity (9). Traditionally, *Turbinariaconoides*has been used to cure children’s fever, as fertilizer, insect repellent, pesticide and antibacterial (10). Phytochemical investigation of Brown alga revealed the presence of steroids, flavonoids and reducing sugars (11). The present investigation was carried out to explore upon antibacterial, antifungal, antiviral and cytotoxic activities with various extract viz. *n*-hexane, cyclohexane, methanol and ethanol:water (1:1) of Brown alga. 

## Experimental

*Algal material*

*Turbinariaconoides*was collected in September 2005 from SalinMunthal, Gulf of Mannar, Bay of Bengal, Ramanathapuram district, Tamil Nadu, India and voucher specimen was deposited at Marine algal research station, Mandapam camp, Tamil Nadu, South India. It was also authenticated by K.Eswaran, Scientist, Marine algal research station, India. Brown alga was air-dried for 4 weeks at room temperature. The dried algal material was coarsely powdered and stored in a polyethylene bag under refrigeration. 

*Extraction*

The powdered *Turbinariaconoides*(1kg) was successively extracted with 2.5 L of *n*-hexane, Cyclohexane, methanol and ethanol:water (1:1), each by maceration with occasional shaking at room temperature for 72 h. The *n*-hexane 1, cyclohexane 2, methanol 3 and ethanol:water (1:1) 4 extracts were concentrated under reduced pressure and kept in desiccator for further investigation. The yields of 1, 2, 3 and 4 were 0.21%, 0.22%, 8.68% and 10.31% w/w, respectively. The chemicals were obtained from Qualigens (GlaxoSmithkline Pharmaceuticals Ltd.), Mumbai, India and Rankem (Ranbaxy Pharmaceuticals), New Delhi, India. 

*Bacteria and fungi*

The extracts were screened against a panel of microorganisms, including *Staphylococcus aureus *subsp. *aureus *(MTCC 737), *Staphylococcus epidermidis *(MTCC 3615), *Escherichia coli *(MTCC 1687), *Psuedomonasaeruginosa*(MTCC 424), *Aspergillusniger*(MTCC 228) and *Candida albicans*(MTCC 183). The investigated microbial strains were procured from the Institute of Microbial Technology, Chandigarh, India. 

*Preparation of inoculum*

Active cultures for screening were prepared by transferring a loopful of cells from the stock to test tubes of nutrient broth for bacteria, yeast peptone dextrose broth for *Candida albicans*and Czapek yeast extract broth for *Aspergillusniger*. Moreover, they were incubated without agitation for 24 h at 37 ºC, 48 h at 30 ºC and 7 days at 30 ºC, respectively as per the guidelines specified by Institute of Microbial Technology, Chandigarh, India. The cultures were diluted with fresh broths to achieve optical densities corresponding to 106 colony-forming units (cfu/mL) for bacteria and 105 spores/mL for fungal strains. 

*Antibacterial and antifungal studies*

Extracts 1, 2, 3 and 4 were dissolved in 100% dimethylsulphoxide (DMSO) at a concentration of 1 mg/mL and used as working stocks. Ampicillin (25 μg) for bacteria, and Clotrimazole (30 μg) for fungi were used as reference agents. Susceptibility test was determined by disc diffusion method (12- 14). The nutrient agar plates were prepared by pouring 15 mL of molten media into sterile petriplates. The plates were allowed to solidify for 5 min, 0.1% inoculum suspension was swabbed uniformly, and the inoculum was allowed to dry for 5 min. The extracts 1, 2, 3 and 4 were loaded on 6 mm discs. The loaded discs were placed on the surface of medium and the extracts were allowed to diffuse for 5 min and the plates were kept for incubation at 37 °C for 24 h for bacteria and 30 °C for 48 h for fungi with yeast peptone dextrose agar and Czepak yeast extract agar media. At the end of incubation, inhibition zones formed around the discs were measured with transparent ruler in millimeters. 

*Determination of minimal inhibitory concentration (MIC)*

A broth dilution susceptibility assay was used for the determination of the MIC (15). Briefly, bacterial strains were cultured overnight at 37 °C in nutrient agar; *Candida albicans*and *Aspergillusniger*were cultured overnight at 30 °C in yeast peptone dextrose agar and Czepak yeast extract agar, respectively. Bacterial and fungal strains were suspended in their corresponding broths to give a final density of 10^6^ and 10^5^ organism/mL respectively. Dilutions of extracts 1, 2, 3 and 4 ranged from 1000 μg/mL to 0.05 μg/mL were prepared in capped tubes. A control was also served; 20 μL from each of the test organisms was used to inoculate the tubes. The tubes were incubated at 37 °C for 24 h for bacteria and at 30 °C for 48 h for fungi. Tubes containing broth (2 mL) were inoculated with organisms and kept at +4 °C in a refrigerator overnight to be used as standards. The MIC was recorded as the lowest concentration at which no microbial growth was observed.

*Viruses and cells*

The origin of the viruses was as the following: herpes simplex virus-1 (strain KOS), herpes simplex virus-2 (strain G), vaccinia virus, vesicular stomatitis virus, herpes simplex virus-1 TK- KOS ACVr ,coxsackie virus B-4, sindbis virus, puntatoro virus, reovirus-1 (ATCC VR-230) and parainfluenza virus-3 (ATCC VR-93) (American Type Culture Collection, Rockville, Md.). The virus stocks were grown in human embryonic lung (HEL) cells (herpes simplex virus-1, herpes simplex virus-2, vaccinia virus, vesicular stomatitis virus and herpes simplex virus-1 TK- KOS ACV^r^ ), human epithelial (HeLa) cells (vesicular stomatitis virus and coxsackie virus B4) and Vero cells (parainfluenza-3 virus, reovirus-1, sindbis virus, coxsackie virus B4, and puntatoro virus).

*Antiviral assays*

Confluent cell cultures in microtiter trays were inoculated with 100 CCID_50_ (1 CCID_50 _corresponding to the virus stock dilution that proved infective for 50% of the cell cultures). After 1 h of virus adsorption to the cells, residual virus was removed and replaced by cell culture medium (eagle minimal essential medium) containing 3% fetal calf serum and various concentrations of the test extracts (200, 100, 40, 20, 10, 4 μg/mL). Viral cytopathogenicity was recorded as soon as it reached completion in the untreated virus-infected cell cultures, *i.e*., at 1 to 2 days for vesicular stomatitis; at 2 days for coxsackie; at 2 to 3 days for herpes simplex types 1 and 2, and vaccinia; and at 6 to 7 days for reo and parainfluenza viruses. brivudin, ribavirin, acyclovir, gancyclovir and (S)-9-(2, 3-dihydroxypropyl) adenine were used as reference agents. Antiviral activity was expressed as minimal inhibitory concentration (MIC_50_) required to reduce virus induced cytopathogenicity by 50% (within the micro tray well) (16). 

*Cytotoxicity*

Although confluent cell cultures had not been infected, they were treated with various concentrations of the test extracts, which were incubated in parallel with the virus-infected cell cultures and examined microscopically at the same time as the viral cytopathogenicity was recorded for the virus-infected cell cultures. A disruption of the cell monolayer, *e.g*. rounding up or detachment of the cells, was considered as evidence for cytotoxicity. Cytotoxicity was expressed as minimal cytotoxic concentration (MCC) required causing a microscopically detectable alteration of normal cell morphology of the confluent cell cultures that were exposed to the test extracts. 

## Results and Discussion

Extracts 2 and 3 were found to be effective against both Gram-positive and Gram-negative organisms. They exhibited a broad array of antibacterial activity at 1mg/mL concentration. The MIC ranges of extracts 2 and 3 were found to be between 27 and 2 μg/mL and 105 and 2.1 μg/mL, respectively. Extract 1 showed activity only against *Escherichia coli *with MIC of 3.4 μg/mL. The result is presented in Table 1.

**Table 1:** Antimicrobial activity of extracts 1, 2, 3 and 4.

**Microorganisms **	Zone of inhibition(mm)*						
	**1 **	**2 **	**3 **	**4 **	**Ampicillin ** **(25μg) **	**Clotrimazole ** **(30μg) **	**DMSO **
*Staphylococcus aureus *subsp. *aureus *	Nl	10	10	Nl	25	ND	Nl
*Staphylococcus epidermidis *	Nl	8	8	Nl	8	ND	Nl
*Escherichia coli *	12	12	9	Nl	16	ND	Nl
*Psuedomonasaeruginosa*	Nl	10	9	Nl	7	ND	Nl
*Aspergillusniger*	16	23	12	Nl	ND	25	Nl
*Candida albicans*	15	17	Nl	Nl	ND	17	Nl

*: values indicate sterile disc diameter (6 mm) and are mean of three replicates

DMSO : Dimethyl sulphoxide

ND : Not determined

Nl : No inhibition

In the antifungal activity, extracts 1 and 2 were effective against both the screened fungi. Extract 1 had MIC of 4.6 and 5.1 μg/mL against *Aspergillusniger*and *Candida albicans*, respectively, whereas extract 2 possessed 0.09 and 3.9 μg/mL. Extract 3 showed activity only against *Aspergillusniger*with MIC of 0.55 μg/mL. The MICs of extracts against tested bacterial and fungal strains are depicted in Table 2.

**Table 2:** Minimum inhibitory concentration (μg/mL) of extracts 1, 2, 3 and 4

**Microorganisms **	**1 **	**2 **	**3 **	**4 **	**Ampicillin **	**Clotrimazole **
*Staphylococcus aureus *subsp*. aureus *	ND	2.0	2.1	ND	0.15	ND
*Staphylococcus epidermidis *	ND	10.3	105	ND	5.0	ND
*Escherichia coli *	3.4	3.0	3.2	ND	0.2	ND
*Psuedomonasaeruginosa*	ND	27.0	13	ND	1.3	ND
*Aspergillusniger*	4.6	0.09	0.55	ND	ND	0.05
*Candida albicans*	5.1	3.9	ND	ND	ND	0.06

ND: Not determined

As prerequisite for antiviral tests, the cytotoxicity of the extracts against virus cells was investigated. A moderate cytotoxicity was observed for extract 2 in HEL cells with MCC at ≥ 20 μg/mL. However, it was found to be non-toxic to HeLa and Vero cells at 100 μg/mL. Extracts 1, 3 and 4 were also non-toxic to all the tested cell lines. Moderate activity ( ≥ 20 μg/mL) was shown by extracts 1 and 2 against all the viruses tested while rest of the extracts were not active ( > 100 μg/mL). The results are shown in Table 3. 

**Table 3:** Antiviral* and Cytotoxicity** of extracts 1, 2, 3 and 4.

**Virus (strain) **	**Cell **	**1 **	**2 **	**3 **	**4 **	**BVDU**a **(μm) **	**Ribavirin ** **(μm) **	**ACV**b **(μm) **	**GCV**c **(μm) **
HSV-2 (G)	HEL	>20	>20	>100	>100	50	>250	0.4	0.032
Vaccinia	HEL	>20	>20	>100	>100	0.4	150	250	>100
Vesicular stomatitis	HEL	>20	>20	>100	>100	>250	>250	>250	>100
HSV-1 (TK-KOS ACVr)	HEL	>20	>20	>100	>100	10	>250	50	4
Cytotoxicity	HEL	100	≥20	>100	>100	>250	>250	>250	>100
								**(S)-DHPA**d**(μm)**	
Vesicular stomatitis	HeLa	>20	>20	>100	>100	>250	30	250	
Coxsackie B_4 _	HeLa	>20	>20	>100	>100	>250	150	>250	
Cytotoxicity	HeLa	100	100	>100	>100	>250	>250	>250	
Parainfluenza-3	Vero	>20	>20	>100	>100	>250	250	150	
Reovirus-1	Vero	>20	>20	>100	>100	>250	>250	250	
Sindbis	Vero	>20	>20	>100	>100	>250	>250	>250	
Coxsackie B4	Vero	>20	20	>100	>100	>250	>250	>250	
Punta Toro	Vero	>20	>20	>100	>100	>250	150	>250	
Cytotoxicity	Vero	100	100	>100	>100	>250	>250	>250	

*Minimum inhibitory concentration (μg/mL) required to reduce virus-induced cytopathogenicity by 50%.

**Minimum cytotoxic concentration (μg/mL) required to reduce to cause a microscopically detectable alteration of normal cell morphology.

Cell lines used: human embryonic lung (HEL), human epithelial (HeLa) and Vero cells. ^a^Brivudin^b^ gancyclovirc a^c^yclovir ^d^ (S)-9-(2, 3-dihydroxypropyl) adenine.

Cyclohexane and *n*-hexane extracts show moderate antiviral activity. Among them, cyclohexane extract possesses moderate cytotoxicity in HEL cells. The extracts only exhibited medium activity, because the bioactive compounds may be present in too low concentrations to show effective antiviral activity. Furthermore, the amount of active constituents present in the Brown alga depends on the geographical distribution, season of collection, climate, and ecological condition at the collection site. 

*n*-hexane extract exhibits inhibitory activity only against *Escherichia coli *and Ethanol: Water (1:1) extract, thus it is ineffective against all the tested organisms. However, cyclohexane and methanolic extracts present a broad spectrum of antibacterial activity, while they are effective against *Staphylococcus aureus *subsp. *aureus*, *Staphylococcus epidermidis*, *Escherichia coli *and *Psuedomonasaeruginosa*. 

Cyclohexane and *n*-hexane extracts show an appreciable antifungal property. Among these two extracts, cyclohexane is more active than *n*-hexane with zone of inhibition of 23 mm and 17 mm against *Aspergillusniger*and *Candida albicans*, respectively. 

Our results indicate that cyclohexane extract of *Turbinariaconoides*has moderate antiviral activity, cytotoxicity and broad spectrum of antibacterial property. This potentiality would seem to support the traditional claim as an antibacterial. It can also be concluded that cyclohexane extract (0.09 μg/mL) is as active as clotrimazole (0.05 μg/mL) against *Aspergillusniger. *The phytochemical characterization of the cyclohexane extract, the identification of the responsible bioactive compounds and the elucidation of the mode of action are necessary. 
